# Filamentous Left Ventricular Mass Mimicking Infective Endocarditis: A Case Report and Review of the Literature

**DOI:** 10.7759/cureus.95042

**Published:** 2025-10-21

**Authors:** Julia Qamar Bilalaga, Khadija Jalal, Mohammed Alkowatli, Muhammed Anwer Rafiq, Hamza Alkowatli, Dania Hammadi

**Affiliations:** 1 Medical School, Gulf Medical University, Ajman, ARE; 2 Cardiology, Rashid Hospital, Dubai, ARE; 3 Internal Medicine, HCA Florida Blake Hospital, Bradenton, USA; 4 Medical School, Royal College of Surgeons in Ireland, Bahrain, BHR

**Keywords:** cardiac mass, echocardiography, infective endocarditis, papillary cardiac fibroelastoma, valve vegetation

## Abstract

Cardiac masses may be detected on echocardiography, and filamentous lesions can resemble infective endocarditis, which may complicate diagnosis and management. Despite advances in imaging, distinguishing true vegetations from non-infectious lesions remains challenging.

We report the case of a 71-year-old man with multiple cardiovascular comorbidities, in whom a mobile, filamentous mass was identified in the left ventricle during routine imaging. Initial concern for infective endocarditis prompted the initiation of empirical antimicrobial therapy. However, further evaluation with transesophageal echocardiography and serial blood cultures did not confirm infection. A multidisciplinary review favored a benign etiology, with features most consistent with papillary fibroelastoma; however, histopathological confirmation was not obtained. Hence, antibiotics were discontinued, and the patient was managed conservatively with clinical and imaging follow-up and remained stable.

This case highlights the overlap between IE and non-infectious cardiac lesions such as benign tumors, thrombi, and valvular excrescences. It underscores the limitations of imaging alone in differentiating these entities and emphasizes the need to integrate clinical presentation and microbiological testing along with the imaging findings. To contextualize this case, an illustrative search of previously published cases within the last 10 years was conducted, and the most relevant cases were selected. A brief review of the literature was performed, outlining reported clinical features, diagnostic strategies, and management approaches in similar presentations.

Cardiac masses can mimic infective endocarditis and pose significant diagnostic challenges. Clinicians should maintain a high index of suspicion for such mimics, as a multidisciplinary, evidence-based approach is essential to avoid misdiagnosis, prevent overtreatment, and ensure appropriate patient care.

## Introduction

Vegetation-like masses on cardiac valves can arise from various etiologies, including thrombus (such as nonbacterial thrombotic endocarditis), benign tumors (e.g., papillary fibroelastoma, myxoma), and, less commonly, malignant tumors (primary sarcoma or metastasis) [1]. The most critical diagnosis to consider is infective endocarditis (IE), a relatively rare but potentially life-threatening infection of the endocardial surface of the heart [2,3]. Cardiac masses detected by echocardiography can arise from diverse etiologies and may mimic IE, creating diagnostic uncertainty and risking overtreatment [4]. Therefore, non-infectious causes must also be carefully evaluated.

Infective endocarditis most frequently affects the mitral valve, followed by the aortic, tricuspid, and pulmonary valves [5,6]. It is a heterogeneous disease with variable clinical manifestations, which can make diagnosis particularly challenging in atypical cases [1]. Although advances in echocardiographic imaging have improved the detection of vegetations, benign structures can appear similar, creating diagnostic uncertainty. Therefore, accurately distinguishing true vegetations from alternative diagnoses is critical to prevent unnecessary antimicrobial therapy and surgical intervention [7].

We present a case of a filamentous left ventricular mass initially treated as infective endocarditis, later reclassified as most consistent with papillary fibroelastoma (PFE). To provide context, we performed an illustrative literature search of reports where cardiac masses were mistaken for infective endocarditis, focusing on diagnostic strategies, clinical features, and outcomes. 

This case emphasizes the diagnostic challenge of differentiating infectious from non-infectious cardiac lesions and highlights the need for an integrated approach combining clinical assessment, microbiological testing, and multimodality imaging to guide accurate diagnosis and management. 

## Case presentation

**Figure 1:**
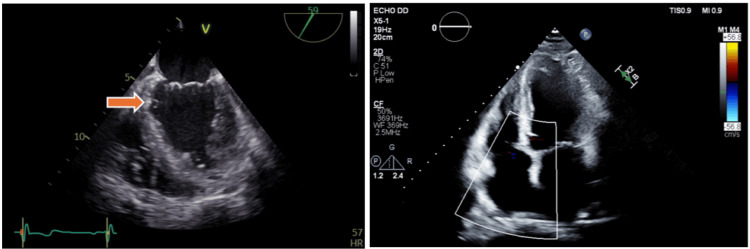
TTE of the Left Ventricle A 12 × 4 mm filamentous mass attached to the interventricular septum just below the mitral valve (orange arrow), suggestive of a mobile intracardiac mass, visualized in both parasternal short-axis and apical four-chamber views. TTE=transthoracic echocardiograph

A 71‑year‑old man with ischemic heart disease (prior CABG with patent LIMA-LAD and SVG-D1, occluded SVG-RCA), type 2 diabetes mellitus, hypertension, aortic sclerosis, and grade 2 diastolic dysfunction was admitted for evaluation of suspected infective endocarditis following an incidental finding on routine transthoracic echocardiography (TTE). Imaging revealed a 12 × 4 mm highly mobile, filamentous echodensity with pedunculated attachment to the inferior interventricular septum just below the mitral valve, raising concern for a possible vegetation, remnant of chordae tendineae, or intracardiac mass (Figure 1).

On review, the patient was asymptomatic: he denied fever, chills, night sweats, chest pain, syncope, recent dental or other invasive procedures, and intravenous drug use. Vital signs were within normal limits, and systemic examination showed no cardiac murmur or peripheral stigmata of infective endocarditis (no Janeway lesions, Osler nodes, splinter hemorrhages, petechiae, or conjunctival hemorrhages). Respiratory and abdominal examinations were unremarkable.

Initial blood tests showed a leukocyte count of 7,200/µL, CRP 4 mg/L, and ESR 15 mm/h (Table 1), all within reference ranges. On the day of admission, the first set of blood cultures grew gram‑positive cocci in one of two bottles, subsequently identified as either *Staphylococcus epidermidis* or *Staphylococcus hominis*; all subsequent cultures remained sterile. The infectious diseases team interpreted the single‑bottle isolate as most likely contamination.

**Table 1:** Laboratory Results CRP = C-reactive protein; ESR = erythrocyte sedimentation rate.

Test	Patient Values	Reference Range	Units
Leukocyte count	7,200	4,000 – 11,000	/µL
CRP	4	< 5	mg/L
ESR	15	0 – 20	mm/h

Given the echocardiographic appearance and the isolated culture result, empiric intravenous antimicrobial therapy was started (ceftriaxone 2 g daily and vancomycin 1 g every 12 hours) with monitoring of vancomycin troughs and renal function, and intravenous fluids to mitigate nephrotoxicity risk.

The cardiology team subsequently reviewed the case and recommended a transesophageal echocardiography (TEE) for further characterization of the mass. TEE revealed a small, filamentous, partly calcified, pedunculated mass attached to the inferior interventricular septum by a short stalk, confirming the TTE findings. There was no evidence of valvular destruction, vegetation, thrombus, intracardiac shunt, or left atrial appendage thrombus. The cardiac valves were structurally intact, showing only mild mitral and tricuspid regurgitation and a sclerotic aortic valve (Video 1).

**Video 1:** TEE Mid-Esophageal Modified Four-Chamber View A mobile, calcified filamentous mass attached to the interventricular septum just below the mitral valve, highlighting its size, mobility, and pedunculated attachment. TEE=transesophageal echocardiography

A multidisciplinary review (cardiology and infectious diseases) applied the Modified Duke-ISCVID criteria; the patient did not meet criteria for definite infective endocarditis because of the absence of clinical features, normal inflammatory markers, and persistently negative serial blood cultures [8]. On TEE, the lesion’s morphology (small size, pedunculated stalk, filamentous surface, and high mobility) favored a benign valvular tumor, most consistent with PFE; however, definitive diagnosis could not be established because histopathological confirmation was not obtained, as the patient declined biopsy.

Considering the lesion’s small size, lack of embolic phenomena, absence of symptoms, and the patient’s comorbidity profile, antibiotics were discontinued after infectious disease review, and a conservative strategy of serial clinical and echocardiographic surveillance was adopted. Repeat TTE at six months showed a stable lesion in size and mobility, and the patient remained asymptomatic on outpatient follow‑up.

## Discussion

This case illustrates the diagnostic challenge of distinguishing IE from other intracardiac masses with overlapping echocardiographic features in modern clinical practice. Our patient, a 71-year-old asymptomatic male, was initially suspected of IE following incidental findings on TTE that closely resembled vegetations, a classical major criterion for IE [8-10].

The initial detection of gram-positive cocci in one of two blood culture bottles prompted empirical antimicrobial therapy in accordance with institutional protocol for suspected IE. However, this isolated finding, with subsequent negative cultures, was later recognized as insufficient microbiological evidence under the Duke-ISCVID criteria and most consistent with contamination, a well-recognized pitfall affecting 2-3% of samples [11]. Such contamination can lead to unnecessary antimicrobial therapy, underscoring the importance of obtaining multiple cultures and interpreting microbiological results in conjunction with clinical and imaging findings [12]. 

While recent modifications have improved diagnostic precision, accurate application still requires nuanced clinical judgment to avoid both over- and underdiagnosis [13]. A key part of an effective diagnostic process for this case includes recognizing the broad differentials for intracardiac masses, which include benign tumors (PFE, myxoma), thrombi, pannus, fibrin strands, and non-infective vegetations [14].

Among these entities, PFE was considered the leading differential diagnosis in this case. The lesion’s echocardiographic characteristics, a small, highly mobile, pedunculated mass with a filamentous surface and a short stalk, closely resembled the typical morphology of PFE. These features, along with its attachment to the interventricular septum just below the mitral valve, were highly suggestive of a benign valvular tumor rather than an infectious vegetation. However, histopathological confirmation could not be obtained, as the patient declined surgical or biopsy intervention. Consequently, the diagnosis remained presumptive, based on multimodal imaging and clinical stability over serial follow-up.

Nonbacterial thrombotic endocarditis (NBTE), also known as Libman-Sacks endocarditis, represents another important differential diagnosis for valvular masses. While it is noninfective in origin, its echocardiographic appearance can resemble other intracardiac lesions such as PFE or vegetations seen in infective endocarditis. NBTE lesions typically occur on the basal or mid-portion of the mitral and aortic valves and may affect both sides of the leaflet, whereas IE vegetations are more often found on the atrial surface of the mitral valve or the ventricular surface of the aortic valve and are associated with valvular destruction or regurgitation. Kovacs et al. (2023) described a middle-aged woman with NBTE of the mitral valve that was initially misdiagnosed as a PFE based solely on transesophageal echocardiographic findings [15], illustrating how noninfective cardiac lesions can closely mimic one another and highlight the limits of imaging alone in establishing a definitive diagnosis. Similarly, in our patient, the distinction between PFE and other non-infective masses was ultimately reliant on clinical context, lesion stability, and absence of embolic or inflammatory complications.

Although major advances in echocardiographic technology, such as three-dimensional and strain imaging, have improved mass detection and characterization, these modalities alone may not always distinguish infectious vegetations from non-infective mimics, as highlighted in prior case reports [14,15]. Even transesophageal echocardiography, while highly sensitive, has limited specificity, reinforcing the necessity of integrating imaging with clinical and laboratory data [16].

In the context of our case, transesophageal echocardiography was particularly valuable in excluding vegetation or thrombus and in defining the lesion’s benign morphology. However, as in similar reports, the absence of tissue diagnosis limited definitive classification, emphasizing the role of longitudinal follow-up in establishing benign behavior over time.

In our patient’s case, invasive interventions such as biopsies were justifiably deferred due to several factors: the mass was small (12 × 4 mm), mobile but non-obstructive, and exhibited benign features on transesophageal echocardiography. There was no evidence of prior stroke, systemic embolization, hemodynamic compromise, or progressive lesion growth, factors typically warranting more aggressive intervention [14]. The significant risks, such as perforation, arrhythmia, and embolization, associated with carrying out an endomyocardial biopsy in an elderly patient with multiple cardiovascular comorbidities, were also considered. Overall, the risk-benefit ratio strongly favored conservative monitoring. More importantly, the patient expressed a preference to avoid invasive procedures after shared decision making, which aligns with the current best practice recommendations for managing asymptomatic, well-characterized cardiac masses [17]. 

Management strategies for cardiac masses are etiology-specific. Keeping in mind the high rates of mortality, confirmed IE mandates prolonged, targeted intravenous antibiotics and consideration of surgery only for refractory infection, hemodynamic failure, or embolic risk [12,18]. Hence, our patient was promptly started on intravenous antibiotic therapy upon arriving at the provisional diagnosis of infective endocarditis. Once IE was ruled out, the antibiotics were discontinued, emphasizing the importance of reevaluation of working diagnoses as new clinical and biochemical data emerge. 

Surgical excision is indicated for large, mobile, or embolic lesions or if malignancy is suspected [19]. In contrast, conservative follow-up and discontinuation of antibiotics are appropriate in asymptomatic, low-risk patients such as ours. In such cases, guidelines recommend periodic echocardiographic surveillance and clinical assessment to monitor for changes in mass size or mobility, or new symptoms such as embolic or arrhythmic events [14]. 

Long-term care for our patient includes regular follow-up at 6 and 12 months with echocardiography to ensure stability of the lesion, consistent with current recommendations. This vigilant strategy enables early detection of progression while minimizing unnecessary interventions.

In conclusion, this case underscores the diagnostic ambiguity between infective and non-infective cardiac masses, particularly PFE and NBTE, in the era of advanced imaging. It highlights the indispensable role of multimodality assessment and multidisciplinary review in differentiating benign from pathological entities, while reinforcing that imaging findings must always be interpreted in the context of microbiological and clinical evidence.

Literature review and case context

To contextualize the present case within the existing literature, we conducted a focused review of previously reported cardiac masses and valvular lesions that mimicked infective endocarditis (IE) both clinically and on imaging. These reports collectively illustrate the diagnostic overlap between benign cardiac tumors, thrombotic lesions, and infective vegetations, which can lead to initial misclassification as IE.

The cases span a broad demographic range from young adults to elderly patients and encompass a variety of pathological entities, including PFE, myxoma, thrombus, and NBTE. A concise summary of representative cases, highlighting patient demographics, lesion location, imaging modalities used, management strategies, and final diagnoses, is presented in Table 2.

**Table 2:** Summary of Reported Cardiac Masses Mimicking Infective Endocarditis TTE = transthoracic echocardiogram; TEE = transesophageal echocardiogram; NBTE: nonbacterial thrombotic endocarditis; PFE= papillary fibroelastoma; MRI = magnetic resonance imaging; CT = computed tomography; PET-CT = positron emission tomography–computed tomography; FDG PET = fluorodeoxyglucose positron emission tomography; MVR = mitral valve replacement

Author/ Year	Age/Sex	Location	Initial Diagnosis	Imaging	Management	Final Diagnosis/Outcome
Yang and Hu (2024) [20]	70/Male	Mitral valve	Infective endocarditis	TTE, TEE	Surgical excision	Cardiac hemangioma; full recovery
Kovacs et al. (2023) [15]	49/Female	Mitral valve	Infective endocarditis	TEE	Conservative	NBTE (Libman-Sacks); embolic stroke
Kim et al. (2020) [21]	24/Male	Left atrium	Infective endocarditis	TTE, TEE	Surgical resection	Cardiac myxoma (Carney complex); full recovery
Collado-Rivera et al. (2021) [22]	79/Male	Mitral valve	Infective endocarditis	TTE, TEE	Surgical resection	PFE; no recurrence
Ramos et al. (2023) [23]	55/Male	Interatrial septum	Infective endocarditis	TEE, FDG-PET	Antibiotics	Right atrial mass; resolved
Marino et al (2025) [24]	65/Female	Right atrium	Infective Endocarditis	TTE, TEE, cardiac MRI, PET-CT	Antibiotic therapy, planned coronary angiography	Right atrial myxoma, exact outcome not specified
Koźma et al. (2019) [25]	61/Female	Pulmonary valve	Infective Endocarditis	TTE, TEE	Surgical excision	PFE; favorable recovery
Zulfa and Habibie (2023) [26]	23/Female	Mitral valve	Infective Endocarditis	TTE, CT	Thrombectomy, MVR	Cardiac myxoma; full recovery
Hammersley et al. (2017) [27]	Middle-aged/F	Left atrium	Infective Endocarditis	TTE, TEE	Surgical excision	Fibrinous mass; full recovery
Fitzgerald et al. (2018) [28]	23/Male	Left atrium	Infective Endocarditis	TTE, TEE	Surgical excision	Atrial myxoma with *Streptococcus viridans* colonization; full recovery

Epidemiology and Patient Demographics

Cardiac masses mimicking infective endocarditis (IE) occur predominantly in adults aged from the 20s through the 80s and affect both males and females. The current case involves a 71-year-old male, consistent with many reports indicating that these masses commonly arise in middle-aged to elderly patients. Most reported cases identified masses located on or near the mitral valve or within the left atrium, consistent with typical sites for benign cardiac tumors such as myxomas and papillary fibroelastomas [15, 20-23, 25-28]. The present case is unique in that the mass was a filamentous lesion attached to the interventricular septum below the mitral valve, an unusual site that broadens the known spectrum of possible cardiac mass locations mimicking IE.

Anatomical Location of Cardiac Masses

Most cases reviewed demonstrated involvement of the mitral valve, left atrium, or interatrial septum [20-28]. Many masses mimicked infectious vegetations on valves, whereas others were intracavitary or located within cardiac chambers [20-28]. This variable anatomical distribution underscores the diagnostic challenges due to overlapping echocardiographic features with IE vegetations.

Initial Clinical Diagnosis

In the majority of reported cases, including this one, the initial diagnosis was infective endocarditis based on echocardiographic visualization of mobile intracardiac masses coupled with clinical suspicion [20-28]. The current case was initially suspected as IE due to the detection of a mobile echodensity and a single positive blood culture. However, systemic infectious symptoms were notably absent. Similar diagnostic uncertainty was reported in other cases, where patients ranged from asymptomatic individuals to those presenting with embolic or constitutional symptoms [20-28].

Diagnostic Imaging Modalities

Transthoracic and transesophageal echocardiography were the primary imaging tools utilized throughout all cases. Advanced imaging modalities such as intraoperative TEE, cardiac MRI, CT, and fluorodeoxyglucose positron emission tomography (FDG-PET) were variably employed to clarify mass characteristics. Despite these technological improvements, echocardiographic features alone were insufficient to confidently differentiate infectious vegetations from tumors, thrombi, or other non-infectious masses, emphasizing the necessity for integrating comprehensive clinical data [14-16, 20-28].

Management Strategies

Management protocols adhered closely to the underlying etiology revealed during evaluation. Cases suspected of IE were initially treated with empirical antibiotics, which were discontinued upon exclusion of infective pathology [12, 18, 20-28]. Surgical excision was the most common definitive intervention for benign tumors, especially in patients with embolic risk or significant hemodynamic compromise [17,19-28]. Conservative management with serial imaging and clinical monitoring was reserved for low-risk, asymptomatic lesions or in patients unsuitable for surgery [14,17,19-28]. The present case exemplified this approach, with prophylactic antimicrobial therapy initially applied and then ceased following diagnostic clarification, followed by close follow-up.

Outcomes and Prognosis

Outcomes reported across cases were generally favorable, with good recovery following surgery and low recurrence rates for benign tumors such as myxomas and fibroelastomas [20-28]. Conservatively managed cases exhibited stability over periods of follow-up without adverse events [20-28]. The mass location and pathology in the current case predict a similarly favorable prognosis, contingent on ongoing surveillance, given the rare septal attachment. These findings support a tailored, multidisciplinary management approach to optimize patient outcomes while minimizing unnecessary interventions.

## Conclusions

Filamentous cardiac masses can mimic infective endocarditis, posing diagnostic challenges. Accurate diagnosis relies on integrating clinical assessment, serial blood cultures, and multimodality imaging like TTE and TEE. Careful interpretation of isolated positive cultures is essential to avoid unnecessary antibiotics. Conservative management with close follow-up is effective for asymptomatic patients with benign-appearing lesions. Multidisciplinary collaboration remains key to optimizing patient outcomes and preventing overtreatment.
